# Should we open the reviewing process

**Published:** 2021-03

**Authors:** Vladimir Mrša

**Affiliations:** Editor-in-Chief of Food Technology and Biotechnology; University of Zagreb, Faculty of Food Technology and Biotechnology, Pierottijeva 6,; HR-10000 Zagreb, Croatia

## Dear reader,

One year after the outbreak of COVID-19 and the rapid global spread of the SARS-CoV2, the virus is still here, people still get infected, many have to be hospitalized and some unfortunately die. We are still afraid of the disease, but we know much more about our enemy and it makes us less insecure. We have learned how to live with the virus. Epidemiologists have told us how to behave to minimize its spread, molecular biologists have explained how the virus enters the body and our cells and what damage it causes, biotechnologists have created the vaccine to protect us from the infection, engineers have built new hospital facilities and equipment, and psychologists have mobilized to help us overcome mental problems caused by the pandemic. Science responded by focusing research and expanding communication as we needed to acquire more knowledge in as short time as possible and to communicate research results among scientists, as well as with authorities and general public. A demand for fast dissemination makes the quality assurance of disseminated data more important than ever. Nature can be regarded as a large puzzle and the role of scientists is to incorporate pieces of knowledge in the overall understanding of how the world around us functions. Inserting a wrong piece not only blurs the puzzle but also misleads other scientists in their efforts to fill in the existing gaps. Therefore, it is our task not only to explore but also to consolidate efforts of the whole scientific community. The crucial part of these efforts is to ensure high quality peer reviewing of research data. Indeed, this may represent the main role of scientific journals today. The mechanism of anonymous peer reviews has been the essence of quality control in most journals for many decades. In the recent years, however, as the number of research papers has multiplied, demanding ever more time and efforts to ensure credibility of published data, journals are seeking innovations in the publishing practices including the reviewing mechanisms. In view of the culture of ‘openness’ in the total research process, open reviews have attracted attention of the scientific publishing community. Although the term ‘open review’ can mean different reviewing practices, in recent discussions it most often means that the names of the reviewers are revealed to authors. The idea of disclosing names of reviewers is not exactly a new one. Over twenty years ago, Nature Neuroscience published an article summarizing advantages and disadvantages of open peer reviews (*1*). Reading this article, one can conclude that the main dilemma is still very much up-to-date. From the point of view of publishers and editors, the main motivation for open review is increased credibility of the journal and generally better quality of reviews that facilitate editors’ decisions which papers to publish and which to reject. High quality reviews also improve final, published versions of manuscripts and diminish the possibility of publishing erroneous, inadequately obtained or manipulated results, or even frauds. The major disadvantage is that every journal has a ’community’ of reviewers some of whom would not feel comfortable to disclose their names. Thus, finding adequate reviewers for an ever-increasing number of papers may become even more complicated and tedious. This may be the main reason why the vast majority of journals remain reluctant in experimenting with the traditional reviewing process.

From the point of view of the authors, the situation in which their papers are evaluated by unknown reviewers while at the same time their names are known to them may seem unfair. It is not easy for authors to accept rejections of their papers based on reviewers’ opinions without knowing their credentials. It should, however, be mentioned that the decision on the fate of the paper is never brought by the reviewers alone, but it is always the responsibility of the editor, who can give authors the opportunity to revise their manuscript regardless of the reviewer’s criticism, or ask for another opinion if the quality of the review is not as high as it should be. The reviewers’ opinions and editor’s decision based on them should be as objective as possible, regardless of whether the reviewers’ names are disclosed or not. On the other hand, a good review can bring valuable improvements to the text, as well as to the quality of authors’ research, and at the same time, by opening the reviewing process and disclosing reviewers’ names with their comments, reviewers would receive due credit, which could be an incentive for them to accept peer reviewing task. Considering this, it seems that the idea of open peer review attracts more attention of the editors than of the authors and the scientific community in general, although in a number of publications dealing with this mode of quality assurance some less obvious advantages are mentioned, like increasing the general discussion on scientific topics, or education of younger researchers (*2*). Anyway, the number of journals that implement, or at least experiment with some kind of open peer reviews is slowly increasing and becoming a trend in modern publishing (*3*). What changes it will bring and which problems of today’s journals it will solve is yet to be seen, but it could reshape the landscape of scientific publishing a great deal. Digital publishing, abolishing printed versions of journals, and online presentation of scientific results have already changed the role of the journal remarkably. From a dissemination vehicle for research data whose creation involved technical editing of the text, quality assurance through peer reviewing, creating the layout, printing, and finally distributing the journal to libraries, it has become an institution whose role is simply to ensure credibility of data contained in a manuscript that could otherwise be processed by the authors themselves and published through an institutional repository. Indeed, what makes world’s top scientific journals so appreciated is the trust of the scientific community that papers published in these journals contain facts that have been subjected to the quality assurance of highest possible scrutiny. If, however, a paper was published together with the accompanying reviews signed by the highly esteemed authorities, wouldn’t it have the same scientific merit, influence and recognition as those published in a repository of a research institute or university? This would certainly simplify the dissemination process and make it cheaper, allowing more research funds to be allocated to acquiring data and less to their publishing. How scientific journals would respond and adjust to the new modes of scientific publishing remains to be seen.
